# Effects of Respiratory Muscle Training on Pulmonary Function in Individuals with Spinal Cord Injury: An Updated Meta-analysis

**DOI:** 10.1155/2020/7530498

**Published:** 2020-02-22

**Authors:** Xiaojun Wang, Na Zhang, Yubin Xu

**Affiliations:** ^1^Thoracic Surgery, Taizhou Central Hospital (Taizhou University Hospital), Taizhou, 318000 Zhejiang, China; ^2^School of Food (Biology) Engineering, Xuzhou University of Technology, Xuzhou, 221018 Jiangsu, China; ^3^Department of Pharmaceutics, Taizhou Central Hospital (Taizhou University Hospital), Taizhou, 318000 Zhejiang, China

## Abstract

**Objective:**

To investigate the pulmonary function responses to respiratory muscle training (RMT) in individuals with tetraplegia and provide a systematic review of the included studies.

**Methods:**

Computerized retrieval of randomized controlled trials (RCT) in PubMed, Embase, and the Cochrane Library on the improvement of respiratory function in patients with spinal cord injury by RMT was conducted until May 2019. Two researchers independently screened the literature, extracted the data, and evaluated the risk of bias in the included studies. Articles were scored for their methodological quality using the Cochrane Collaboration risk of bias assessment tool.

**Results:**

Sixteen studies were identified. A significant benefit of RMT was revealed for five outcomes: force vital capacity (FVC, WMD: -0.43, 95% CI -0.84 to -0.03, *P* = 0.037), vital capacity (VC, WMD: -0.40, 95% CI -0.69 to -0.12, *P* = 0.037), vital capacity (VC, WMD: -0.40, 95% CI -0.69 to -0.12, *P* = 0.037), vital capacity (VC, WMD: -0.40, 95% CI -0.69 to -0.12, *P* = 0.037), vital capacity (VC, WMD: -0.40, 95% CI -0.69 to -0.12, *P* = 0.037), vital capacity (VC, WMD: -0.40, 95% CI -0.69 to -0.12,

**Conclusion:**

Our findings demonstrate that RMT can effectively improve spinal cord injury pulmonary function of the patient, which is marked by increasing respiratory strength, function, and endurance. Limited by the quantity and quality of the included studies, the above conclusion needs to be verified by more high-quality studies.

## 1. Introduction

The injury of the cervical and upper thoracic spinal cord impairs the respiratory function involved in inspiratory and expiratory muscles [1], specifically, diaphragm, intercostal muscles, accessory respiratory muscles, and abdominal muscles, which are also main sources of morbidity and mortality of SCI due to the pulmonary complications, such as atelectasis or pneumonia, both in the short and long terms after the injury [2, 3]. In clinic, most individuals with tetraplegia have decreased capacity to get air into the lungs and reduced ability to cough to remove secretions. Initially, following SCI, the ventilatory response is characterized by early and progressive dynamic lung hyperinflation, which is associated with an elevated work of breathing and an increased severity of dyspnea. In the long term, reduced lung volumes and decreased chest wall compliance become the major manifestation [4]. In addition, some previous study has reported in patients with SCI the existence of airway obstruction [5]. As such, respiratory dysfunction secondary to muscle weakness severely affects the quality of life including exercise tolerance [6]. How will pulmonary function be improved? Recent studies have focused on methods for improving respiratory function. Respiratory muscle training (RMT) is a therapeutic technique that involves the enhancement of respiratory muscle and becomes a clinical focus.

RMT aims at improving respiratory muscle strength and endurance by using methods such as impedance load and threshold pressure load. Early studies on the effect of RMT on healthy adults and athletes indeed provide convincing evidence increasing cardiopulmonary function and further supporting motor performance [7]. However, we should be cautious about judging whether it makes sense, especially in SCI patients. Various RMT improving respiratory strength and endurance include the use of both resistive and threshold trainers and singing training that stimulate inspiratory and expiratory muscles effectively and synchronously. Although a multitude study has pointed out that RMT has positive significance to improve the respiratory function of SCI patients, there is little information about their management. Therefore, the aim of this study was to conduct a randomized controlled trial to compare the effects of respiratory resistance on respiratory function in individuals with complete tetraplegia.

## 2. Methods and Materials

### 2.1. Search Strategy

The electronic databases PubMed, Embase, Cochrane Library, CNKI, Wanfang Data, and VIP were searched using the terms “inspiratory”, “respiratory”, “breathing exercises”, “exercises”, “SCI”, “spinal cord injuries”, “paraplegia”, and “quadriplegia”. The last research was on May 23, 2019. Additional publications were also searched via scanned reference lists of articles identified in the initial searches.

### 2.2. Inclusion and Exclusion Criteria

The inclusion criteria were as follows: (1) randomized controlled trials (RCTs); (2) adult patients (18 years or older) who have been diagnosed with spinal cord injury; (3) compared respiratory muscle training intervention with placebo, usual, or routine care; and (4) outcome measures including lung function (lung function for force vital capacity (FVC), vital capacity (VC), maximal voluntary ventilation (MVV), and forced expiratory volume in 1 second (FEV_1_)) or respiratory muscle strength (maximum static inspiratory pressure (MIP), maximum static expiratory pressure (MEP)).

The exclusion criteria were as follows: (1) review, abstract, case report, or conference literature; (2) duplicate publication; and (3) the relevant data not reported.

### 2.3. Data Extraction and Quality Assessment

Included data were independently extracted by two investigators using a standardized form. The inconsistency conflicts were resolved by discussion with a third researcher. The following data was extracted: the first author, publication year, country, sample size, intervention, and outcome measures. The Cochrane Collaboration risk of bias tool was used to assess the methodological quality of included studies.

### 2.4. Statistical Analyses

Statistical analyses were performed using Stata 12.0 software (StataCorp, College Station, TX) and RevMan 5.3 (The Cochrane Collaboration, Software Update, Oxford, UK). For continuous data, the pooled standardized mean differences (SMDs) and their corresponding 95% confidence intervals (95% CIs) were used to assess the strength. *P* < 0.05 was considered as statistically significant. We used the Chi-squared test and *I*^2^ test to assess heterogeneity. A fixed effects model was adopted when *P* > 0.10 or *I*^2^ < 50%. Otherwise, the random effects model was adopted. Sensitivity analysis was performed to evaluate the stability of results. Sensitivity analysis was performed by excluding one study to assess the influence of any single study. Publication bias was assessed via Begg's funnel plot and Egger's linear regression test.

## 3. Results

### 3.1. Characteristics of the Included Studies

**Figure 1:**
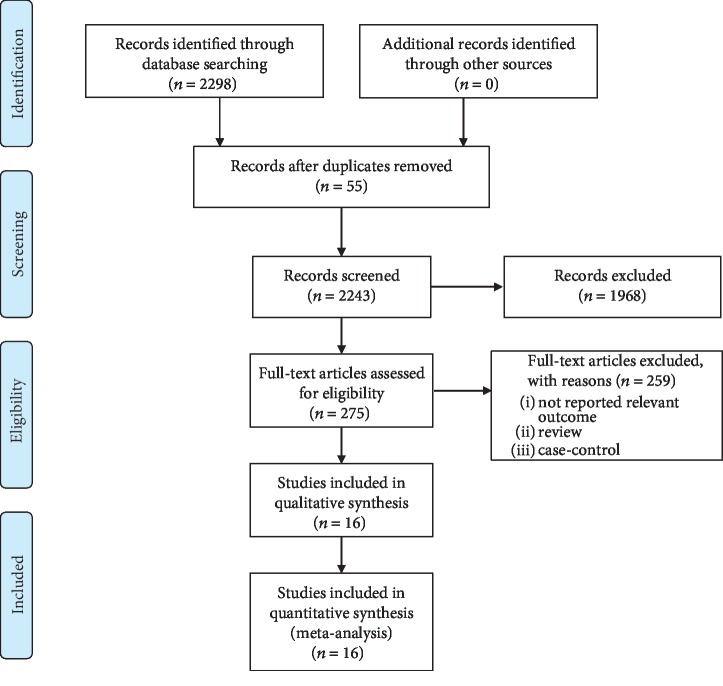
Flow diagram of the article selection process.

2298 studies were identified through electronic database search. 55 studies were further selected after removing duplicates. 275 relevant articles were further identified after screening titles and abstracts. 11 articles were excluded because they failed to meet the inclusion criteria. As a result, 16 articles [3, 6, 8–21] (237 cases and 211 controls) were included in this review (Figure 1). Of the 16 included trials, the published year is between 1990 and 2018. Nine studies [3, 6, 8, 12, 14, 16–19] used inspiratory or expiratory muscle resistance training. Seven studies [9–11, 13, 15, 20, 21] used training inspiratory and expiratory muscle. Three studies assessed two different interventions and a control condition, such as Mueller et al.'s [15] design which is divided into inspiratory resistance training and isocapnic hyperpnea training compared with placebo, Litchke et al. [11] who compared two different interventions (concurrent flow resistance and concurrent pressure threshold) with usual care, and Kim et al.'s [10] studies which compared integrating training and respiratory muscle training with placebo (Table 1).

### 3.2. Methodological Quality

**Figure 2:**
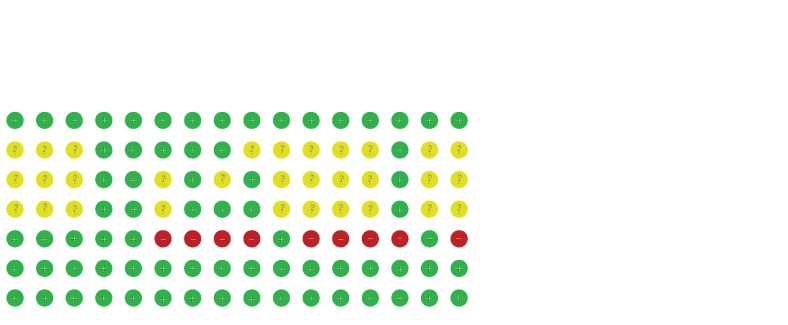
Risk of bias of included studies.

The methodological quality of included studies was assessed using the Cochrane Collaboration risk of bias tool (Figure 2). Among the 16 included studies, 16 studies reported the randomization procedure. Six studies clearly reported the allocation concealment. Six articles mentioned the blinding procedures. In the incomplete outcome data section, nine studies were considered “high risk” due to participants who did not complete the trial.

### 3.3. Pooled Analyses

#### 3.3.1. VC

**Figure 3:**
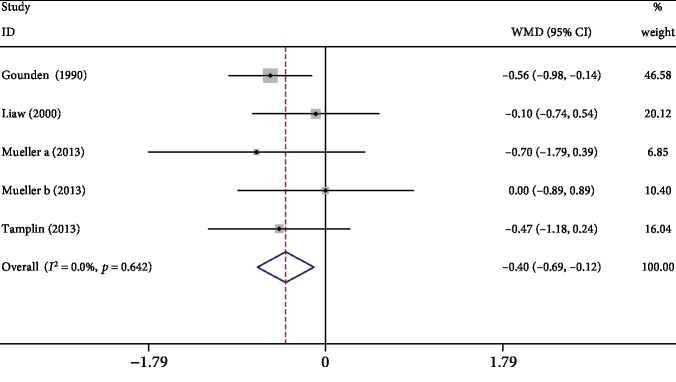
Forest plot of meta-analysis results for vital capacity.

Four studies [12, 15, 17, 20] reported respiratory exercise's effects on VC. Meta-analysis showed that compared to the control, respiratory exercise significantly improved VC (WMD: -0.40, 95% CI -0.69 to -0.12, *P* = 0.006, *I*^2^ = 0%; Figure 3).

#### 3.3.2. FVC

**Figure 4:**
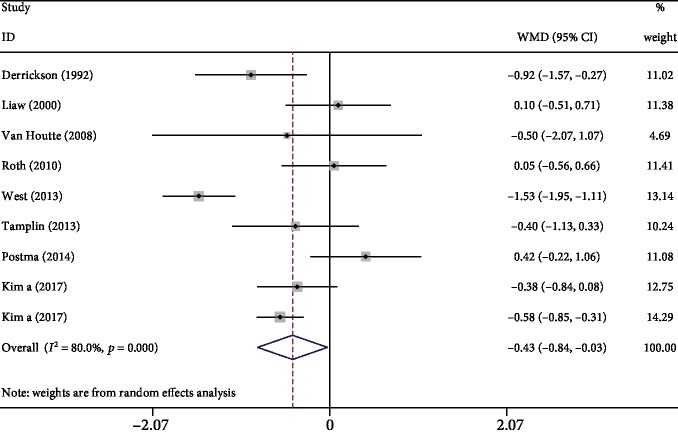
Forest plot of meta-analysis results for force vital capacity.

Compared to the control, respiratory exercise showed superior effects on FVC in 8 studies [3, 6, 8, 10, 12, 18, 20, 21] (WMD: -0.43, 95% CI -0.84 to -0.03, *P* = 0.037, *I*^2^ = 80%; Figure 4). Although significant heterogeneity among the included trials was detected, sensitivity analysis was performed by removing West's study, and the results remained unchanged.

#### 3.3.3. FEV_1_

**Figure 5:**
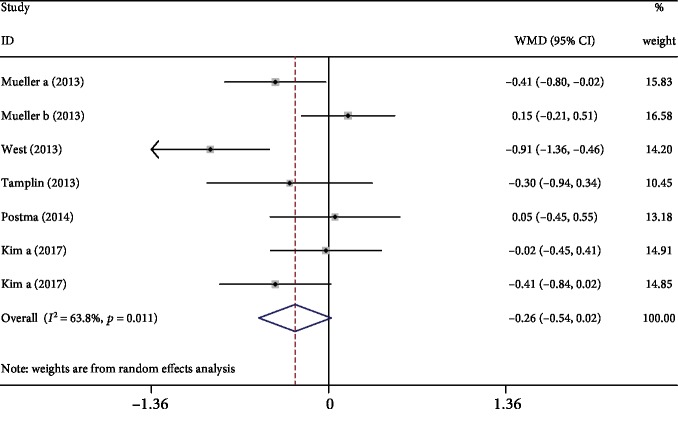
Forest plot of meta-analysis results for forced expiratory volume in 1 second.

Five studies [6, 10, 15, 18, 20] provided available data for FEV_1_. Meta-analysis showed that respiratory exercise did not influence FEV_1_ when compared with the control (WMD: -0.26, 95% CI -0.54 to -0.02, *P* = 0.07, *I*^2^ = 63.8%; Figure 5). Sensitivity analysis indicated that the conclusion was not changed after excluding West's study. Removing West's study, *I*^2^ decreased from 63.8% to 28.7%.

#### 3.3.4. MEP

**Figure 6:**
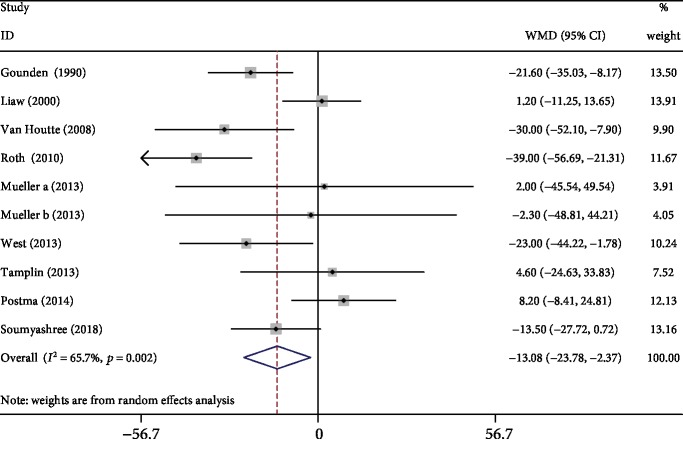
Forest plot of meta-analysis results for maximum static expiratory pressure.

Nine studies [3, 6, 12, 15, 17–21] reported MEP data. The pooled analysis demonstrated that respiratory exercise was associated with significantly improved MEP when compared with the control (WMD: -13.08, 95% CI -23.78 to -2.37, *P* = 0.017, *I*^2^ = 65.7%; Figure 6). We performed sensitivity analysis by removing Roth's study, and the results remained unchanged.

#### 3.3.5. MVV

**Figure 7:**
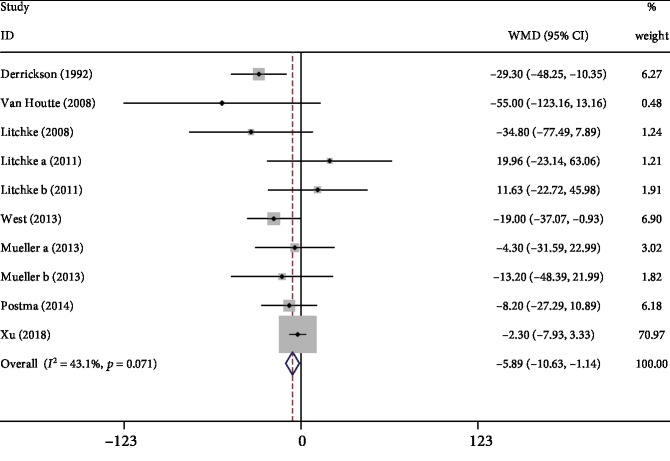
Forest plot of meta-analysis results for maximal voluntary ventilation.

Eight studies [6, 8, 9, 11, 13, 15, 18, 21] reported data on MVV when respiratory exercise is compared to the control group. Meta-analysis showed that the respiratory exercise had a significant superior effect (WMD: -5.89, 95% CI -10.63 to -1.14, *P* = 0.015, *I*^2^ = 43.1%; Figure 7).

#### 3.3.6. MIP

**Figure 8:**
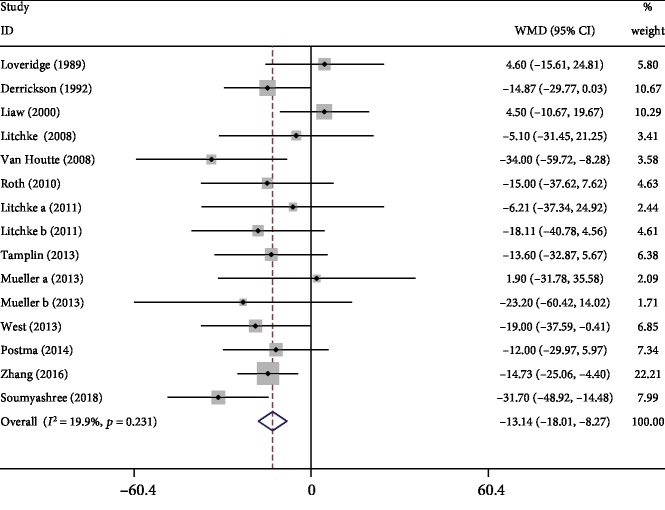
Forest plot of meta-analysis results for maximum static inspiratory pressure.

Thirteen studies [3, 6, 8, 11–16, 18–21] assessed the effects of respiratory exercise on improvement of MIP. Meta-analysis showed that the respiratory exercise significantly improved MIP compared with the control group (WMD: -13.14, 95% CI -18.01 to -8.27, *P* < 0.001, *I*^2^ = 19.9%; Figure 8).

### 3.4. Publication Bias

**Figure 9:**
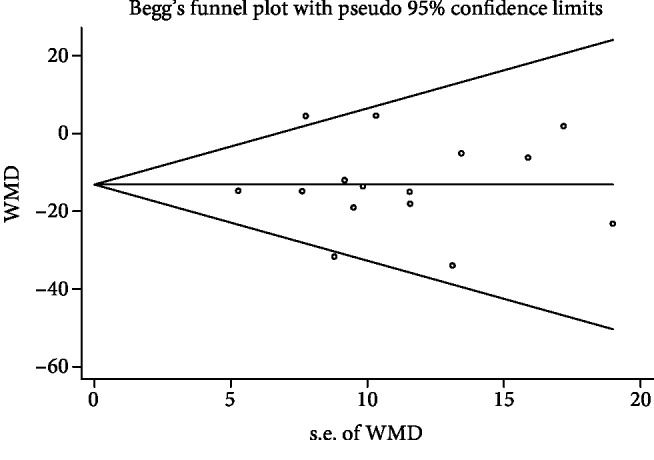
Funnel plot of meta-analysis results for maximum static inspiratory pressure.

The publication bias was assessed based on MIP, and results indicated that there was no significant publication bias (Egger's *P* value = 0.991; Begg's *P* value = 0.729; Figure 9).

## 4. Discussion

This systematic review and meta-analysis focused on the effect of RMT on the full range of pulmonary function measured in tetraplegia. Not only is body movement and sensory dysfunction caused by spinal cord injury but it also adversely affects respiratory function in the long term, leading to chronic hypoxia of the whole system [22]. SCI patients with respiratory dysfunction usually appear with breathing difficulties, cough, and sputum weakness [23]. Especially in daily activities, it is difficult to complete function tasks due to cardiopulmonary insufficiency, which certainly increases the risk of death.

It is well known that the spirometry and lung volume studies in persons with tetraplegia and high levels of paraplegia have demonstrated restrictive dysfunction due to neuromuscular weakness characterized by a significant reduction of VC, FEV_1_, MVV, etc. [24]. As the main respiratory muscle, diaphragmatic muscle is innervated by the phrenic nerve in the cervical spinal cord levels 3-5. Once the diaphragm is denervated, respiratory dysfunction occurs. The higher the level of injury, the more significantly pulmonary function parameters are reduced [5]. In this study, RMT was shown to be more effective in improving VC, FVC, MIP, and MVV. Actually, MEP was also improved by RMT in the previous research [25].

MIP reflects the combined inspiratory strength of all inspiratory muscles, while MEP for expiratory muscle. Both the MIP and the MEP are very sensitive to the absolute lung volume, and they are the most commonly used, reliable, and noninvasive index to evaluate respiratory muscle function. Long-term training is bound to improve the strength and endurance of respiratory muscles [2], among which the diaphragm is the most obvious and important. Although speculative, a reduction in abdominal compliance following RMT may enhance diaphragm contractility. In addition to the increase of muscle strength and endurance mentioned above, it can also improve the compliance of chest and alveolar elasticity and lung ventilation [26]. These training methods also have the potential to increase the strength and effectiveness of voluntary independent cough, decrease the amount of retained secretions, and thereby reduce the occurrence of pneumonia and other causes of respiratory morbidity [3]. In general, RMT yields some benefit to SCI patients to some degree except pulmonary function, such as central haemodynamics and exercise capacity.

It has to be pointed out that studies currently published about RMT have a small number, and training methods are not particularly specific. In addition, the limitations of this study include the following: (i) Although 16 studies were included in this review, the sample size was relatively small in every study. More large sample studies need to confirm this conclusion. (ii) Participants were lost or incomplete in some studies, which may contribute to a high risk of bias. (iii) The SCI level of participants was different; we could not determine the proper level of RMT.

In summary, the results from this meta-analysis provide more solid evidence that RMT improves lung function and respiratory muscle strength in people with SCI through increasing FVC, VC, MVV, MIP, and the MEP index. In addition, more large-scale RCTs are needed to further explore long-term efficacy and optimal treatment parameters of RMT. In addition, the mechanism of enhanced respiratory muscle pump through RMT is promising and warrants further investigation.

## Figures and Tables

**Table 1:** Characteristics of the studies.

Author & year	Country	Gender (M/F)	Sample size (T/C)	Intervention	Control	Treatment duration	Main outcome
Gounden, 1990	South Africa	32/8	20/20	Resistive EMT	Usual care	8 w	VC, MEP
Xu, 2018	China	95/5	50/50	Respiratory resistance training	Usual care	2 m	FVC, FEV_1_, MVV
Van Houtte, 2008	Belgium	12/2	7/7	Normocapnic hyperpnea training	Sham	8 w	FVC, PEM, MVV
Litchke, 2011	USA	NR	9/7	(a) Concurrent flow resistance (b) Concurrent pressure threshold	Usual care	9 w	MVV, MIP
Mueller, 2013	Netherlands	18/6	16/8	(a) Isocapnic hyperpnea training (b) Inspiratory resistance training	Sham	8 w	MVV, MIP, VC, FEV_1_, MEP
Derrickson, 1992	USA	9/2	6/5	Resistive IMT	Usual care	7 w	FVC, MVV,
West, 2014	UK	9/1	5/5	Resistive IMT	Sham	6 w	FVC, MVV, FEV_1,_ MEP
Litchke, 2007	USA	9/0	4/5	Respiratory resistance training	Usual care	10 w	MVV, MIP
Liaw, 2000	China	16/4	10/10	Resistive IMT	Usual care	6 w	FVC, MIP, FEV_1,_
Loveridge, 1989	Canada	NR	6/6	Resistive IMT	Usual care	8 w	FVC
Postma, 2014	Netherlands	35/5	19/21	Resistive IMT	Usual care	8 w	MVV, MIP, FVC, FEV_1_
Roth, 2010	USA	22/7	16/13	Resistive EMT	Sham	9 w	FVC, MIP, FEV_1_
Tamplin, 2013	Australia	NR	13/11	Therapeutic singing training	Music appreciation	12 w	VC, FVC, MIP, FEV_1_
Kim, 2017	Korea	22/15	25/12	(a) Integrating training (b) Respiratory muscle training	Sham	8 w	FVC, FEV_1_
Zhang, 2016	China	27/11	19/19	Resistive IMT	Usual care	4 w	MIP
Soumyashree, 2018	India	22/5	15/12	Resistive IMT	Usual care	4 w	MEP, MIP

EMT: expiratory muscle training; IMT: inspiratory muscle training; FVC: force vital capacity; VC: vital capacity; MVV: maximal voluntary ventilation; FEV_1_: forced expiratory volume in 1 second; MIP: respiratory muscle strength; MEP: maximum static expiratory pressure; M: male; F: female; T: treatment; C: control.
